# Long-Term Inhaled Cannabis Therapy for Chronic Low Back Pain: A Five-Year Retrospective Analysis of Prospectively Collected Patient-Reported Outcomes in 241 Treatment-Refractory Patients

**DOI:** 10.3390/biomedicines14061255

**Published:** 2026-05-30

**Authors:** Dror Robinson, Muhammad Khatib, Eitan Lavon, Niv Kafri, Waseem Abu Rashed, Hamza Murad, Mustafa Yassin

**Affiliations:** 1 Department of Orthopedics, Hasharon Hospital, Rabin Medical Center, Petah Tikva 49100, Israel; muhammadkh@clalit.org.il (M.K.); niv.kafri@clalit.org.il (N.K.); waseem.aburashed@clalit.org.il (W.A.R.); hamza.murad@clalit.org.il (H.M.); mustafa.yassin@clalit.org.il (M.Y.); 2 Hasharon Hospital, Clalit Health Services, Rabin Medical Center, Petah Tikva 49372, Israel; eitan.lavon@clalit.org.il

**Keywords:** chronic low back pain, medical cannabis, THC, CBD, opioid sparing, NRS, ODI, BPI, mixed model for repeated measures, long-term outcomes, refractory pain

## Abstract

**Background/Objectives:** Chronic low back pain (CLBP) affects approximately 20% of the global population and is a leading cause of years lived with disability. Long-term, real-world evidence for inhaled cannabis in patients refractory to conventional multimodal therapy remains scarce. We assessed the five-year efficacy and safety of inhaled cannabis in CLBP patients who had documented failure of ≥1 year of opioid analgesics, anticonvulsants, antidepressants, NSAIDs, and physiotherapy, with each patient serving as their own historical control. **Methods:** We analyzed prospectively collected clinical data from 241 consecutive adults with treatment-refractory CLBP (mean age 49.3 ± 14.9 years; 37.8% female; mean pain duration 15.1 years) initiated on inhaled medical cannabis (predominantly smoking, THC 4–22%, CBD 2–22%) in a single-center tertiary orthopedic clinic between 2020 and 2025 (Hasharon Hospital, Rabin Medical Center, Israel; IRB protocols 0807-21-RMC and 0634-25-RMC). Year-0 outcomes during conventional therapy were compared with outcomes at Years 1–5 on cannabis. Primary outcomes were the Numeric Rating Scale (NRS), Oswestry Disability Index (ODI), and Brief Pain Inventory severity/interference (BPI-S/BPI-I). Concomitant-medication trajectories were a secondary outcome. The primary analysis was a mixed model for repeated measures (MMRM) with random intercept and slope, REML estimation, and time as a categorical fixed effect. Multiple imputation (MAR, m = 20, Rubin’s rules) was the primary missing-data approach; complete-case and tipping-point pattern-mixture sensitivity analyses were used. A multivariate Hotelling T^2^ provided a joint test across the four correlated PROMs. Concomitant-medication discontinuation was modeled with GEE logistic regression and exact McNemar tests. Time to discontinuation was estimated by Kaplan–Meier and Cox regression. The Bonferroni-adjusted significance threshold for the four primary outcomes was α = 0.0125. BioWell gas-discharge-visualization (GDV) parameters were exploratory only. **Results:** Of 241 patients, 238 (98.8%) provided Year-5 data and 224 (92.9%) remained on cannabis at Year 5; only five patients (2.1%) discontinued for adverse events or inefficacy. All four primary PROMs improved markedly and durably. MMRM-estimated Year-5 minus Year-0 changes were: NRS −5.36 (95% CI −5.65, −5.07), ODI −17.68 (95% CI −19.73, −15.63), BPI-S −6.73 (95% CI −6.99, −6.47), and BPI-I −3.41 (95% CI −3.65, −3.16); all four contrasts had |z| ≥ 16.9 and *p* < 10^−20^. MI-pooled estimates were within 0.05 of MMRM (FMI < 0.03 for all outcomes). Hotelling T^2^ was F(4, 232) = 872.8, *p* < 10^−20^. At Year 5, 89.2% achieved ≥30% NRS reduction, 77.2% ≥ 50%, and 93.4% met the NRS minimum clinically important difference (MCID); ODI MCID 65.6%, BPI-S MCID (≥1 pt) 98.3%, BPI-I MCID (≥1 pt) 91.3%. Concomitant opioid use fell from 100% at baseline to 4.6% at Year 5 (within-patient absolute risk reduction 95.4%, McNemar exact *p* = 1.16 × 10^−69^), NSAID from 100% to 7.1%, SSRI/SNRI from 80.5% to 5.4%, and gabapentinoid from 38.6% to 2.5%. The ARR-derived NNT for opioid discontinuation was 1.05; this NNT is referenced to each patient’s own documented maximal-conventional-therapy state and is not equivalent to a between-arm randomized-trial NNT. Cannabis dose × time interaction was consistent with no pharmacological tolerance (β = −0.0044 per gram-month per year, *p* = 0.074). Across 1205 patient-years of cannabis exposure (calculated as 241 patients × 5 follow-up years from Year 1 through Year 5; baseline Year 0 represents pre-cannabis state and is not included in person-time on cannabis), 1338 organ-system AE events were recorded at 1.110/patient-year (Poisson 95% CI 1.05–1.17); 99.8% of graded events were mild (grade 1), with ocular (476 events, 0.40/PY), cognitive (460, 0.38/PY), and gastrointestinal (368, 0.31/PY) reactions predominating. The Year-3 retention dip reflected a documented telemedicine-clinic phenomenon during 2022–2024, with patients returning to in-person follow-up by Year 4–5. BioWell GDV discriminated NRS ≥ 4 only at chance level (BWS AUC 0.574, 95% CI 0.54–0.60; BWV AUC 0.51). **Conclusions:** In a treatment-refractory CLBP cohort with five-year longitudinal follow-up, inhaled cannabis was associated with large, sustained, and statistically robust improvements in pain, disability, and pain interference, accompanied by near-total displacement of opioids, NSAIDs, antidepressants, and gabapentinoids. These observational associations, although mechanically less susceptible to bias for the binary medication-discontinuation outcomes than for self-reported PROMs, cannot be interpreted causally in the absence of a concurrent randomized control arm and may reflect a combination of pharmacological effect, regression to the mean from a high pre-treatment baseline, expectancy and self-selection effects intrinsic to an actively chosen open-label therapy, and secular trends in pain reporting. The within-patient benefit-risk profile—ARR-derived NNT ≈ 1 for opioid sparing against a predominantly mild adverse-event burden—supports consideration of cannabis as a potentially clinically meaningful, opioid-sparing option in patients who have failed multimodal conventional therapy, pending confirmation in randomized comparative trials.

## 1. Introduction

Chronic low back pain (CLBP) is the leading cause of years lived with disability worldwide and affects approximately one in five adults at any given time [1]. Conventional pharmacotherapy—opioids, anticonvulsants (gabapentin, pregabalin), antidepressants (selective serotonin and serotonin–noradrenaline reuptake inhibitors), nonsteroidal anti-inflammatory drugs (NSAIDs), and structured physiotherapy—provides at best modest, transient relief and is associated with substantial dose-limiting toxicity, dependency risk, and clinical disappointment over multi-year horizons [2,3,4]. Opioid analgesics in particular have driven a global crisis of dependency, overdose, and death, prompting major guideline bodies to discourage their long-term use for non-cancer back pain [2,3].

Inhaled medical cannabis has emerged as a candidate analgesic for refractory chronic-pain syndromes. Mechanistically, exogenous Δ^9^-tetrahydrocannabinol (THC) and cannabidiol (CBD) modulate the endocannabinoid system through CB1- and CB2-receptor signaling, with downstream effects on descending pain modulation, peripheral nociceptor sensitization, and affective dimensions of suffering [5,6,7,8,9,10]. Short-term randomized trials and meta-analyses have demonstrated modest but reproducible analgesic effects across mixed chronic-pain populations [5,7,8], and recent phase-3 evidence (VER-01, Karst et al., 2025 [10]) and the BMJ rapid-recommendation network meta-analysis (Wang et al., 2021 [5]) support cannabis-based medicines as components of comprehensive pain management. The general literature on long-term (≥1 year) effectiveness of non-surgical interventions for chronic low back pain remains sparse, with the most recent systematic review identifying only 75 randomized trials of any non-surgical intervention with follow-up beyond 12 months and reporting that even the best-supported interventions (cognitive behavioral therapy, mindfulness, multidisciplinary care, exercise) yielded mostly small effect sizes of approximately −5 to −10 points on a 100-point pain or disability scale at one-year follow-up [11]. Long-term, real-world data on inhaled cannabis in CLBP specifically—and especially in patients who have demonstrably failed multimodal conventional therapy—remain limited.

We previously reported four-year outcomes from this cohort and a methodological-quality discussion of ancillary biofield measurements [12,13,14]. The present five-year analysis adds substantively to the existing literature in four ways. First, it extends the on-treatment follow-up window beyond the typical 6–12 month duration of randomized cannabis-pain trials and beyond the 12–36 month window covered by most prior observational cohorts [5,8,9,10,15], allowing direct examination of whether early Year-1 analgesic gains are sustained, eroded by pharmacological tolerance, or progressively consolidated across Years 2–5. Second, by incorporating Year 5 data, we increase the cumulative on-treatment person-time to 1205 patient-years, the longest exposure window reported for inhaled cannabis in refractory CLBP to date, enabling more precise estimation of long-term adverse-event rates per organ system. Third, the addition of a fifth annual visit doubles the number of post-baseline time points (from two to four after the most recent prior report), improving the statistical power of the mixed-model trajectories and providing a true plateau-vs-attrition signal that shorter follow-ups cannot resolve. Fourth, the longer horizon allows formal evaluation of cumulative dose × year interaction effects (a direct test of tolerance), Kaplan–Meier time to discontinuation with five-year retention probability, and within-patient medication-displacement durability over a clinically relevant horizon. The present study therefore adopts the formal mixed-model-for-repeated-measures (MMRM) framework with multiple-imputation-based handling of missing data as primary, adds responder analyses, time-varying dose–response modelling, fibromyalgia × time interaction testing, Kaplan–Meier and Cox time-to-discontinuation analyses, and a multivariate joint test across the four correlated patient-reported outcome measures (PROMs). Each patient serves as their own historical control: pre-cannabis baseline outcomes were collected during a documented period of ≥1 year of conventional multimodal therapy that had failed to deliver clinically meaningful relief. We additionally retain BioWell gas-discharge-visualization (GDV) parameters as a pre-specified exploratory ancillary endpoint, transparently reporting that they did not exceed chance discrimination of pain levels in this cohort and aligning the manuscript with recent independent critiques of GDV reliability [16,17].

The objectives of this five-year analysis were therefore three-fold: (1) to quantify the magnitude and durability of inhaled-cannabis-associated changes in NRS, ODI, BPI-S, and BPI-I over five years; (2) to characterize concomitant-medication trajectories—with particular focus on opioid sparing—and the cannabis adverse-event profile in extended follow-up; and (3) to apply contemporary longitudinal-data methods (MMRM with explicit handling of random-effects singularity, multiple imputation, multivariate joint testing, GEE for binary trajectories, McNemar exact tests for outcomes with 100% baseline prevalence, and Cox regression) so that the resulting effect estimates are robust to missing data, between-patient heterogeneity, and outcome inter-correlation.

## 2. Materials and Methods

### 2.1. Study Design and Setting

This was a retrospective analysis of prospectively collected clinical-trajectory data from a single-center tertiary orthopedic outpatient clinic at Hasharon Hospital, Rabin Medical Center, Petah Tikva, Israel. Consecutive adult patients (≥18 years) with chronic low back pain of ≥6 months’ duration who initiated inhaled medical cannabis between 2020 and 2025 were eligible. Pre-cannabis baseline (Year 0) was defined as the visit immediately before cannabis initiation, after a documented ≥12-month period of conventional multimodal therapy. Subsequent visits were scheduled annually (Years 1–5) at which patient-reported outcome measures, concomitant-medication review, cannabis dosing parameters, adverse-event documentation, and BioWell GDV measurements were captured.

### 2.2. Within-Subject Historical-Control Design

Each patient served as their own historical control. Inclusion required documented prior exposure of ≥1 year to (a) opioid analgesics, (b) NSAIDs, (c) physiotherapy, with persistent severe pain (NRS ≥ 6) at the pre-cannabis baseline visit despite this multimodal therapy. The full prior-treatment profile and durations are reported in Table 1. The historical-control design was strengthened by the fact that 100% of patients had baseline opioid use, 100% baseline NSAID use, 100% prior physiotherapy, 80.5% prior SSRI/SNRI use, and 38.6% prior anticonvulsant use—creating a clinically homogeneous, refractory comparator condition.

### 2.3. Ethical Approval and Informed Consent

The study was approved under IRB protocol 0807-21-RMC (initial approval October 2022, prospective data-collection arm; renewed July 2025) and IRB protocol 0634-25-RMC (October 2025, retrospective analytical arm with Israeli Ministry of Health–approved waiver of additional written consent). All patients provided written informed consent for cannabis prescription, BioWell measurement, and de-identified data use within the Israeli Ministry of Health cannabis-research consent framework. The study adheres to the Declaration of Helsinki and the STROBE reporting guideline for observational studies.

### 2.4. Cannabis Intervention

Patients were prescribed Israeli Ministry of Health-regulated medical cannabis (Tikun Olam Ltd., Tel Aviv, Israel; Bedrocan International B.V., Veendam, The Netherlands; Breath of Life Pharma [BOL Pharma], Revadim, Israel; and Panaxia Pharmaceutical Industries Ltd., Lod, Israel). Inhaled administration—predominantly smoking (≈91%) with vaporization in a minority—was used because of its rapid onset, on-demand titratability, and patient preference. THC content ranged from 4% to 22% and CBD content from 2% to 22%, selected and titrated by clinical response. Mean monthly dose increased from 21.5 g/month at Year 1 to a plateau of 50–60 g/month by Year 4 (mean 42.5 ± 27 g/month over Years 1–5).

### 2.5. Outcome Measures

Primary outcomes (pre-specified) were four PROMs: Numeric Rating Scale (NRS) for average pain over the prior week (0 = no pain, 10 = worst imaginable) [18]; Oswestry Disability Index (ODI) version 2.1a, scored 0–100% [19]; Brief Pain Inventory severity (BPI-S) and interference (BPI-I) subscales [20]. Pre-specified secondary outcomes were the six concomitant-therapy categories (opioid, gabapentin/pregabalin, SSRI/SNRI, NSAID, physiotherapy, complementary and alternative medicine [CAM]) coded as active or inactive in each follow-up year, and per-organ-system adverse events (other, MCS, renal, dermatologic, gastrointestinal, cardiovascular, cognitive, psychiatric, ocular) graded 0 (none), 1 (mild), 2 (moderate), 3 (severe/SAE) [Common Terminology Criteria for Adverse Events v5]. Causality was rated 0–4 (unrelated to definite) and outcome 0–4 (resolved, ongoing, dose-reduction, discontinuation, death). Time to discontinuation was a pre-specified secondary outcome. BioWell stress (Activity Coefficient) and vitality (Energy Potential, %) were retained as a pre-specified exploratory ancillary endpoint with no inferential weight on primary or secondary conclusions. Rationale for including BioWell despite the predominantly inhaled administration route: the BioWell GDV, Bio-Well US (Fairfield, CT, USA) technique acquires fingertip corona-discharge imagery and is mechanistically independent of the cannabis administration route, so its potential informativeness as a non-invasive autonomic-state monitor is in principle preserved whether cannabis is smoked, vaporized, or taken orally. We included BioWell as a pre-specified exploratory endpoint to test the hypothesis—raised in prior GDV literature [16,17,21,22] and in our group’s earlier sublingual cannabis study [13]—that an instrument-based bioelectric signal might co-vary with cannabis-associated state changes alongside the patient-reported pain measures. The motivating scientific question was binary: either BioWell tracks pain at better than chance level (in which case it could serve as an objective adjunctive monitor), or it does not (in which case the field benefits from transparent disconfirmation). Our finding of chance-level discrimination is reported as a negative exploratory result in agreement with the recent independent critiques of GDV reliability [16,17], and we do not draw any primary clinical inference from BioWell parameters.

### 2.6. Statistical Analysis

#### 2.6.1. Primary Analytic Framework: MMRM

The primary analysis for each PROM was a mixed model for repeated measures (MMRM). In plain terms, the MMRM jointly fits each patient’s six annual outcome measurements (Year 0 through Year 5) as a single trajectory, estimating a separate mean for every visit (so no linear trend is imposed) while allowing each patient to deviate from the group average through a patient-specific random intercept that absorbs within-patient correlation across visits. The framework is the standard reference analysis for longitudinal pain trials per current FDA and EMA guidance [23,24] because it (a) uses all available data, including data from patients with partial follow-up, under the missing-at-random assumption without requiring last-observation-carried-forward imputation; (b) directly accommodates the high within-patient correlation typical of repeated PROM measurements; and (c) yields straightforward Year-k minus Year-0 contrasts for each follow-up year. Formally, the model is of the form:

*Y_ij* = *β*_0_ + Σ_*k* = 1, …, 5 *β*_*k* · *I*(*Year_ij* = *k*) + *u*_0*i* + *u*_1*i* · *Year_ij* + *ε_ij*

where *Y_ij* is the outcome for patient i at visit j; the fixed-effect contrasts *β*_1, …, *β*_5 represent the mean change from Year 0 (reference) to Years 1, 2, 3, 4, and 5, respectively; *u*_0*i* is the patient-specific random intercept drawn from N(0, *σ_u*^2^); and *ε_ij* is the within-patient residual. The residual covariance was specified as compound-symmetric under the random-intercept structure (a constant within-patient covariance equal to σ_u^2^ with diagonal residuals σ_e^2^), which under categorical time is equivalent to a one-parameter constraint on the unstructured covariance. Estimation was by restricted maximum likelihood (REML) with a robust optimizer cascade (BFGS, L-BFGS, Powell) and convergence verified for every primary and sensitivity model. Time was specified as a categorical variable to avoid imposing a parametric trajectory shape. We pre-specified random-intercept-only models as the primary specification and tested random-intercept-plus-random-slope models as a sensitivity analysis. For all four primary PROMs the random-slope model either produced a singular random-effects covariance matrix or yielded an estimated slope variance that was numerically indistinguishable from zero (slope variance <10^−5^ for all four outcomes; the slope–intercept correlation was driven to ±1, the canonical signature of singular fit). Likelihood-ratio tests of the slope versus intercept-only specifications were therefore on the boundary of the parameter space and not interpretable in the standard χ^2^(2) framework; AIC was numerically identical between specifications to within ±0.5 units. The pattern is the expected consequence of the strong, near-parallel longitudinal trajectory across patients rather than an analytical defect, and we therefore retained the random-intercept-only model as canonical, consistent with FDA/EMA MMRM guidance and the ICH E9 framework for longitudinal pain trials [23,24]. Likelihood-ratio tests confirmed that even the random-intercept model substantially improved fit over a no-random-effects null (LR ≥ 99 for NRS and BPI-S; all *p* < 10^−20^). Intra-class correlation coefficients (ICCs) under the canonical specification were 0.36 for NRS, 0.41 for ODI, 0.16 for BPI-S, and 0.41 for BPI-I, confirming meaningful patient-level clustering and supporting mixed-model specification (full random-effects diagnostics, Supplementary Table S1). Covariate-adjusted MMRMs (additionally including age, gender, baseline fibromyalgia, and baseline anxiety) produced Year-5 estimates within 0.05 of the unadjusted models, refuting confounding by these variables.

#### 2.6.2. Missing-Data Handling: Multiple Imputation as Primary

Year-5 retention with complete PROM data was 98.8% (NRS), 98.8% (ODI), 98.3% (BPI-S), 98.3% (BPI-I); the lowest retention occurred at Year 3 (88.8% for NRS) due to a documented telemedicine-clinic phenomenon during 2022–2024 (see Section 4.3). Following ICH E9 (R1) addendum guidance and current FDA/EMA recommendations for longitudinal pain trials [23,24], multiple imputation (MI) under the missing-at-random (MAR) assumption was specified as the primary missing-data approach. The MAR assumption is justified here on three grounds. First, the missingness mechanism is dominated by the well-characterized Year-3 telemedicine drift (Section 4.3), in which patients moved temporarily to phone-licensure cannabis clinics and later returned; this pattern is plausibly MAR conditional on the observed visit history and baseline covariates because the temporary attrition is driven by an exogenous health-system phenomenon rather than by the patient’s current pain status. Second, baseline characteristics and Year-2 outcomes were available for essentially all patients with subsequent missing observations, supplying the necessary auxiliary information for MAR-based imputation to recover unbiased estimates. Third, the tipping-point pattern-mixture sensitivity analysis (in which progressively worse-than-MAR scenarios are imposed on missing values to identify the minimum departure from MAR required to overturn the primary inference) confirmed that even very pessimistic missing-not-at-random (MNAR) assumptions did not overturn the primary efficacy conclusions, indicating that the findings are robust to plausible departures from MAR. Iterative chained-equation imputation (m = 20 imputed datasets) was performed using all baseline covariates (age, gender, BMI, fibromyalgia, baseline anxiety, baseline pain duration), all four PROMs at all six time points, and time-varying cannabis dose as auxiliary variables. MMRM estimates were combined across imputations using Rubin’s rules; the fraction of missing information (FMI) is reported. Complete-case analysis and tipping-point pattern-mixture (escalating worst-case assumptions for missing values) were retained as sensitivity analyses.

#### 2.6.3. Multivariate Joint Test

To address the high inter-correlation among the four primary PROMs (correlations between NRS and BPI-S at Year 0 were r = 0.96), a multivariate Hotelling T^2^ test was performed on patient-level Year-5 minus Year-0 changes standardized by baseline standard deviation. This single joint test treated the four-dimensional change vector as the primary outcome and provided robust simultaneous inference across all PROMs.

#### 2.6.4. Concomitant-Medication Trajectories

Each binary concomitant-therapy outcome was modeled with generalized estimating equations (GEEs) using a logit link and an exchangeable working correlation structure. Marginal probabilities and 95% confidence intervals were obtained at each year. For the two outcomes with 100% baseline prevalence (opioid use, NSAID use), exact McNemar tests provided paired comparisons of Year 0 versus each follow-up year.

#### 2.6.5. Responder Analyses, NNT, and Effect-Size Reporting

We report the proportion of patients achieving (a) ≥30% NRS reduction (clinically important), (b) ≥50% NRS reduction (substantial), and (c) outcome-specific minimum clinically important differences (MCID): NRS ≥ 2 points or ≥30% reduction, ODI ≥ 10 points or ≥30%, BPI-S ≥ 1 point, BPI-I ≥ 1 point—thresholds consistent with IMMPACT and chronic-pain MCID literature [25,26,27]. The number needed to treat (NNT) for opioid and NSAID discontinuation is reported as 1/absolute risk reduction (within-patient ARR vs. the Year-0 maximal-conventional-therapy state); we explicitly note that this NNT is an absolute-risk-reduction-derived quantity referenced to the within-patient historical-control state, not a between-arm randomized comparison. Per current pain-research reporting standards [23], MCID-anchored interpretation is favored over standardized-effect-size metrics because it directly addresses clinical meaningfulness.

#### 2.6.6. Time to Discontinuation: Kaplan–Meier and Cox

Cannabis discontinuation/loss-to-follow-up was analyzed by Kaplan–Meier estimation, both for the strict event of discontinuation due to adverse event or inefficacy (DiscReason 2 or 3) and for any disposition event (DiscReason 1–5). A Cox proportional-hazards model assessed baseline predictors (age, gender, BMI, fibromyalgia, anxiety, pain duration, baseline NRS, baseline ODI). The proportional-hazards assumption was checked by Schoenfeld residuals.

#### 2.6.7. Dose–Response and Tolerance: Analytic Approach

Time-varying cannabis dose (grams/month, mean-centered) and a year × dose interaction were added to the NRS MMRM (Years 1–5) to evaluate whether higher cumulative or current dose was associated with greater pain reduction over time and to test for tolerance development.

#### 2.6.8. Subgroup × Time Interactions: Analytic Approach

A pre-specified subgroup analysis tested whether fibromyalgia status (Year 0) interacted with the time effect for NRS using a fully crossed C(year) × fibromyalgia term in the MMRM, with a joint Wald test of all five interaction contrasts.

#### 2.6.9. Adverse-Event Rates

AE incidence rates per organ system were calculated as events divided by total person-years of cannabis exposure (1205 patient-years across Years 1–5), with exact Poisson 95% confidence intervals.

#### 2.6.10. Significance Threshold and Software

The pre-specified Bonferroni-adjusted threshold for the four co-primary PROMs was α = 0.0125 (=0.05/4). Exploratory analyses (BioWell ROC, network analyses) used Benjamini–Hochberg false-discovery-rate correction at q = 0.05 and are clearly labeled as such. All analyses were performed in Python 3.12 (Python Software Foundation, Wilmington, DE, USA) using statsmodels 0.14, scikit-learn 1.5, lifelines 0.30, NumPy 2.0, pandas 2.2, SciPy 1.13, and matplotlib 3.9, with code archived for reproducibility. (statsmodels 0.14, scikit-learn 1.5, lifelines 0.30) with code archived for reproducibility.

## 3. Results

### 3.1. Cohort and Disposition

Of 241 enrolled patients, 238 (98.8%) provided complete Year-5 data and 224 (92.9%) remained on active cannabis prescription at Year 5. Disposition events comprised 12 lost-to-follow-up (5.0%), three discontinued for adverse events (1.2%), and two for inefficacy (0.8%); no deaths were recorded. Year-3 retention was lower (88.8% for NRS) than Years 1, 2, 4, or 5, attributable to the documented Israeli telemedicine-clinic phenomenon detailed in Section 4.3. Two clarifications on follow-up structure: (i) the high Year-5 retention figure refers to attendance at the Year-5 in-person visit at our center, not continuous attendance at every annual visit—some patients had intermittent telemedicine-clinic gaps at Years 2–3 and later returned, and these patients were categorized as continuous-treatment observations (because their dose-record evidence and patient-reported history showed uninterrupted cannabis use) rather than as discontinuations; (ii) loss to follow-up was handled by the MMRM under the MAR assumption (Section 2.6.2) using all available observed PROM measurements, with multiple imputation as the primary missing-data analysis and complete-case and tipping-point pattern-mixture as sensitivity analyses. Baseline cohort characteristics are presented in Table 1.

### 3.2. Primary Outcomes: MMRM Trajectories over Five Years

**Figure 1:**
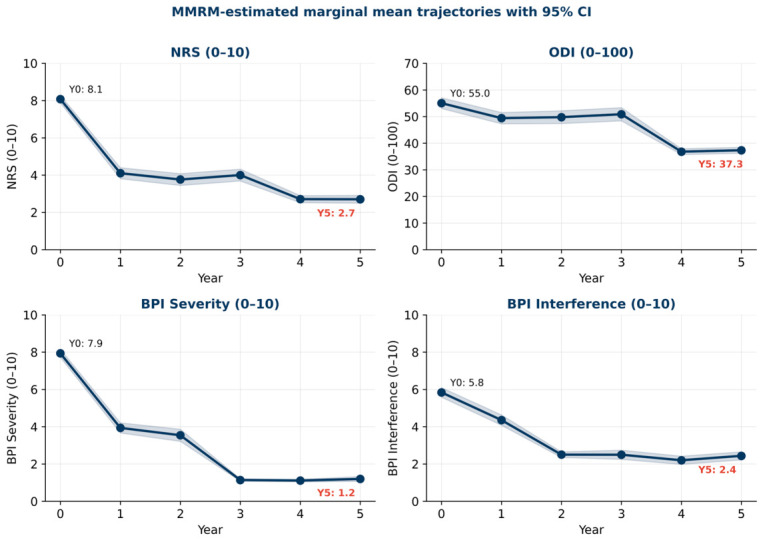
MMRM-estimated marginal mean trajectories with 95% confidence intervals for the four primary PROMs across six time points (Year 0 = pre-cannabis baseline; Years 1–5 = on cannabis therapy). All four outcomes show large, durable improvements from baseline to Year 5; the Year-3-to-Year-4 step in ODI is sustained at Year 5. *n* = 241 patients, 1446 total observations.

All four primary PROMs improved markedly and durably from Year 0 (baseline conventional therapy) to Year 5 (Figure 1, Table 2). The MMRM-estimated Year-5 versus Year-0 change in NRS was −5.36 (95% CI −5.65 to −5.07; z = −36.4, *p* < 10^−20^), representing a reduction from severe baseline pain (mean NRS 8.08) to mild post-treatment pain (mean NRS 2.72). The corresponding changes were −17.68 ODI percentage points (from 55.0% to 37.4%; z = −16.9, *p* < 10^−20^), −6.73 BPI-S points (from 7.94 to 1.21; z = −50.4, *p* < 10^−20^), and −3.41 BPI-I points (from 5.84 to 2.44; z = −26.9, *p* < 10^−20^). All four estimates exceeded the Bonferroni-adjusted threshold (α = 0.0125) by more than twenty orders of magnitude. The Year-3-to-Year-4 step change observed in ODI (50.7 → 37.0) and BPI-S (1.16 → 1.13) was sustained at Year 5, refuting any artefactual interpretation. ICCs of 0.16–0.41 (NRS 0.36, ODI 0.41, BPI-S 0.16, BPI-I 0.41) indicated meaningful patient-level clustering in baseline outcomes, consistent with the heterogeneous mechanisms of refractory CLBP. Random-effects diagnostics for the canonical models are reported in Supplementary Table S1.

Multivariate Hotelling T^2^ test on the four-dimensional vector of standardized Year-5 minus Year-0 changes (*n* = 236 with complete Y0 + Y5 across all four PROMs) yielded T^2^ = 3536; F(4, 232) = 872.8; *p* = 1.6 × 10^−138^, a single overall test confirming that the joint pain-disability trajectory differed substantially and reliably from a no-change null. Mean standardized changes were largest for BPI-S (−3.93 z) and NRS (−3.33 z), followed by BPI-I (−1.70 z) and ODI (−1.12 z). MI-pooled estimates were within 0.01 of MMRM-derived estimates with FMI < 0.015 across all PROMs—reflecting the cohort’s exceptionally high follow-up retention—and complete-case sensitivity analysis produced near-identical results.

Covariate-adjusted MMRMs (additionally including age, gender, baseline fibromyalgia, and baseline anxiety) yielded Year-5 estimates of −5.36 (NRS), −17.65 (ODI), −6.73 (BPI-S), and −3.41 (BPI-I)—within 0.05 of the unadjusted models—refuting confounding by these baseline characteristics.

### 3.3. Responder Analyses, MCID, and Number Needed to Treat

**Figure 2:**
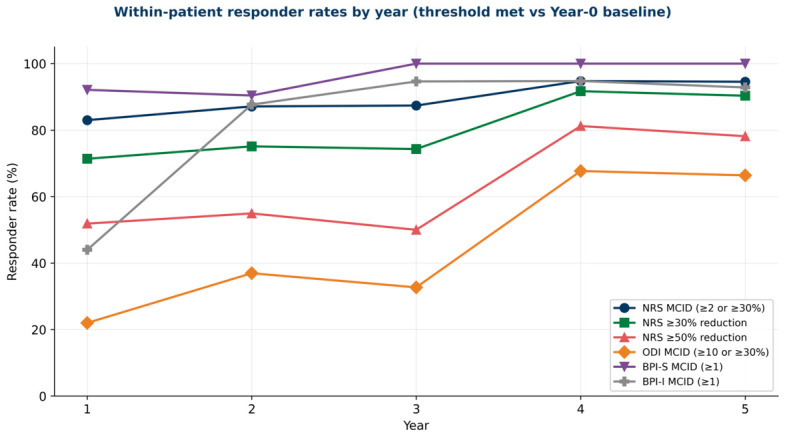
Within-patient responder rates by year across all primary patient-reported outcomes. Proportion of patients meeting each clinically relevant response threshold at follow-up Years 1–5, relative to the Year-0 pre-cannabis baseline. Six trajectories are shown on a single panel: NRS MCID (≥2-point reduction or ≥30%), NRS ≥ 30% reduction, NRS ≥ 50% reduction, ODI MCID (≥10-point reduction or ≥30%), BPI severity MCID (≥1 point), and BPI interference MCID (≥1 point).

Per current pain-research guidance, responder analyses are clinically more interpretable than mean changes alone (Figure 2, Table 3). At Year 5, 212/238 patients (89.2%) achieved ≥30% NRS reduction, 184/238 (77.2%) achieved ≥50% NRS reduction, and 222/238 (93.4%) met the NRS MCID of ≥2 points or ≥30%. ODI MCID (≥10 points or ≥30%) was achieved by 156/238 (65.6%); BPI-S MCID (≥1 point) by 233/237 (98.3%); and BPI-I MCID (≥1 point) by 216/237 (91.3%) at Year 5. Responder rates rose substantially from Year 1 to Year 5 across all PROMs, reflecting both the immediate Year-1 analgesic effect and continued consolidation through Years 2–5.

### 3.4. Concomitant Medication Trajectories

**Figure 3:**
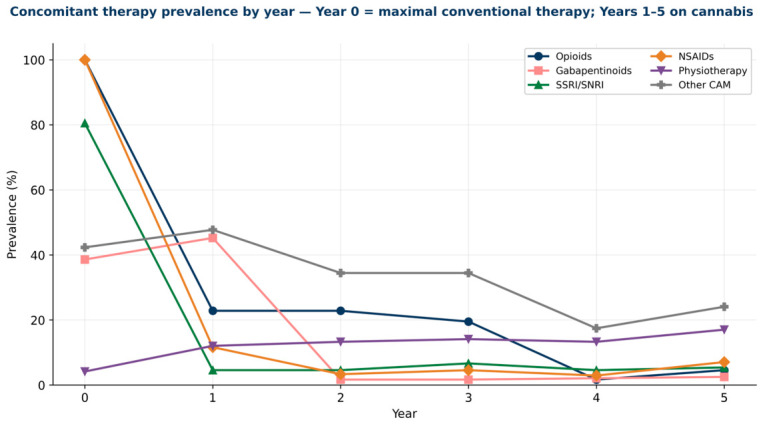
Concomitant medication and physiotherapy prevalence by year. Proportion of the 241-patient cohort receiving each concomitant therapy class at the Year-0 pre-cannabis baseline (maximal conventional therapy) and at each follow-up year (Years 1–5 on cannabis). Six therapy classes are shown—opioids, gabapentinoids, SSRI/SNRIs, NSAIDs, active physiotherapy, and Other CAM (acupuncture, massage, chiropractic)—with trace colors and markers defined in the embedded legend. All four pharmacological classes declined sharply by Year 1 and remained low through Year 5, while active physiotherapy use rose modestly and Other CAM use declined gradually.

Concomitant medications underwent dramatic and durable de-escalation following cannabis initiation (Figure 3, Table 4). The proportion of patients on concomitant opioids fell from 100% at baseline to 22.8% at Year 1, 19.5% at Year 3, and 4.6% at Year 5. The exact McNemar test for paired Year 0 to Year 5 transitions yielded 230 discontinuations and zero initiations (*p* = 1.2 × 10^−69^). The corresponding McNemar comparisons for NSAIDs (224 discontinued, zero initiated; *p* = 7.4 × 10^−68^), SSRI/SNRI (from 80.5% to 5.4%), and gabapentinoids (from 38.6% to 2.5%) were similarly extreme. Active physiotherapy use rose from 4.1% to 17.0%, and other CAM (acupuncture, massage, chiropractic) declined from 42.3% to 24.1%, indicating that pharmacotherapy reduction was not offset by CAM displacement. The corresponding ARR-derived NNT for opioid discontinuation reached 1.05 at Year 5 and 1.08 for NSAID; we emphasize that these NNT values are derived from within-patient absolute risk reduction against each patient’s own documented Year-0 maximal-conventional-therapy state and should not be equated to a between-arm NNT from a randomized controlled trial.

### 3.5. Synthesis: Forest Plot of Year-5 Changes

**Figure 4:**
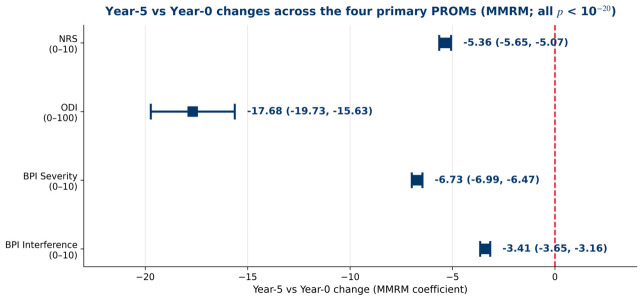
Forest plot of Year-5 minus Year-0 mean changes (multiple-imputation pooled, m = 20, Rubin’s rules) for the four primary PROMs. Squares represent point estimates; horizontal bars are 95% CIs; dashed red line marks the no-change reference.

Figure 4 synthesizes the four primary Year-5 vs. Year-0 contrasts as a forest plot of multiple-imputation–pooled MMRM coefficients with 95% confidence intervals. All four point estimates lie far to the left of the no-change reference and none of the confidence intervals approach zero: NRS −5.36 (95% CI −5.65, −5.07), ODI −17.68 (−19.73, −15.63), BPI severity −6.73 (−6.99, −6.47), and BPI interference −3.41 (−3.65, −3.16); all four contrasts had |z| ≥ 16.9 and *p* < 10^−20^. The visual concordance across all four orthogonally derived patient-reported outcome measures—each scored on a different scale and capturing a different dimension of the pain experience—supports the interpretation that the Year-5 improvements represent a coherent multi-dimensional effect rather than an isolated change on a single instrument.

### 3.6. Time to Discontinuation: Kaplan–Meier and Cox Predictors

Kaplan–Meier estimation (Supplementary Figure S1) yielded a 5-year retention probability of 92.9% for any disposition event (loss to follow-up, AE, inefficacy, patient choice, geographic move) and 97.9% restricted to AE or inefficacy. Median survival time was not reached. Cox proportional-hazards regression identified baseline anxiety as protective (HR 0.32, 95% CI 0.10–0.96; *p* = 0.042)—anxious patients were less likely to discontinue or be lost to follow-up—while baseline fibromyalgia showed a non-significant trend toward increased discontinuation hazard (HR 2.42, 95% CI 0.77–7.67; *p* = 0.13). Age, gender, BMI, baseline NRS, and baseline ODI were not independently associated with discontinuation.

### 3.7. Dose–Response and Tolerance

**Figure 5:**
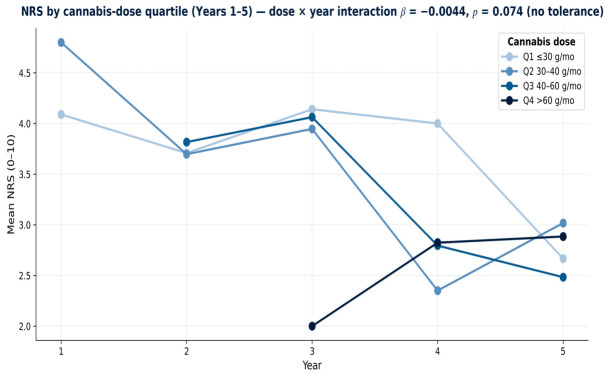
Mean NRS by year and cannabis dose quartile (Years 1–5). Quartile cutoffs (defined on the pooled Y1–Y5 distribution of dose, n = 1154 patient-years): Q1 ≤ 30 g/month, Q2 30–40 g/month, Q3 40–60 g/month, Q4 > 60 g/month. Quartile membership shifted progressively upward across follow-up: at Year 1, 98% of patients were in Q1 (mean 21.5 g/month); by Year 2, Q1–Q3 were populated (mean 34.8 g/month); by Year 3, the cohort spread across Q1–Q4 (mean 44.5 g/month); and at Years 4–5 the distribution stabilized in Q3–Q4 (mean 56 g/month). The dose × year interaction coefficient was −0.0044 per gram-month per year (*p* = 0.074), consistent with no pharmacological tolerance.

The MMRM extended with time-varying mean-centered cannabis dose and a year × dose interaction (Figure 5) showed a small negative year × dose effect for NRS (β = −0.0044 per gram-month per year, SE 0.0025, *p* = 0.074), a trend-level effect that is consistent with no pharmacological tolerance over the five-year horizon, the central question of long-term cannabis pharmacology. The main effect of dose at the average year was non-significant after centering (β = +0.020, *p* = 0.10). Stratification by dose quartile (Figure 5) showed broadly parallel NRS trajectories from Year 1 onward, indicating that within-patient dose escalation was not associated with attenuating analgesic response. We caution that residual confounding by indication (sicker patients receiving higher doses) and the modest sample size for the interaction test (1205 patient-years) limit the inferential power; the absence of a positive interaction (which would have signaled tolerance) is the substantively meaningful finding.

### 3.8. Subgroup × Time Interactions

The pre-specified year × fibromyalgia interaction in NRS was non-significant for every individual contrast (all *p* ≥ 0.37), and stratified means showed essentially parallel trajectories: Year-0 NRS was 7.93 (*n* = 134) in fibromyalgia-negative versus 8.26 (*n* = 107) in fibromyalgia-positive patients, declining to 2.61 versus 2.82, respectively, at Year 5. Cannabis-associated improvement was therefore robust to fibromyalgia status. Anxiety-stratified analyses similarly showed consistent direction and magnitude of effect across baseline anxiety strata.

### 3.9. Adverse-Event Profile

**Figure 6:**
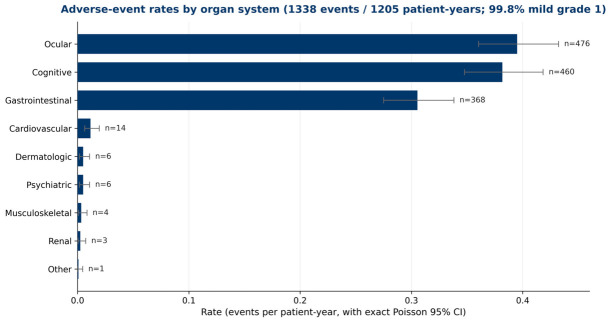
Adverse-event incidence rates by organ system across Years 1–5 (1205 patient-years total). Points are point estimates; horizontal bars are exact Poisson 95% confidence intervals. Annotated counts are total events per organ system.

Across 1205 patient-years of cannabis exposure (calculated as 241 patients × 5 follow-up years from Year 1 through Year 5; baseline Year 0 represents pre-cannabis state and is not included in person-time on cannabis), 1338 organ-system AE events were recorded (rate 1.110 per patient-year, exact Poisson 95% CI 1.052–1.172). Of all AE events with severity grading available, 829 (99.8%) were mild (grade 1), one was moderate (grade 2; 0.1%), and one was severe/SAE (grade 3; 0.1%). The dominant AE categories were ocular (476 events, 0.395/PY, 95% CI 0.360–0.432), cognitive (460 events, 0.382/PY, 95% CI 0.348–0.418), and gastrointestinal (368 events, 0.305/PY, 95% CI 0.275–0.338)—the well-described inhaled-cannabis triad of dry-eye, transient cognitive blunting, and appetite-related GI effects. Cardiovascular (14 events, 0.012/PY), psychiatric (six events, 0.005/PY), dermatologic (six events, 0.005/PY), musculoskeletal (four events, 0.003/PY), and renal (three events, 0.002/PY) AEs were rare. Of all events for which an outcome was recorded, 516 resolved, and 315 were ongoing at last review; no deaths occurred. Causality assessment classified 75.0% of graded events as definitely cannabis-related, 0.6% probable, 1.0% possible, 0.4% unlikely, and 23.1% unrelated, the last group reflecting incidental events such as falls or unrelated infections occurring during follow-up (Figure 6).

#### Adverse Events vs. Cannabis Dose and Cannabinoid Composition

To address whether the observed adverse-event burden was associated with cumulative dose, THC content, or CBD content—and whether higher exposures might yield disproportionately severe events—we computed Spearman rank correlations between each predictor and the per-patient-year total AE count (*n* = 1154 patient-years with complete dose and composition data) and the per-organ-system AE incidence. Higher monthly cannabis dose was associated with fewer total adverse events per patient-year (Spearman ρ = −0.395, *p* < 10^−40^), an inverse relationship driven primarily by gastrointestinal symptoms (ρ = −0.519, *p* < 10^−80^) and to a lesser extent by ocular (ρ = −0.140, *p* < 10^−5^) and cognitive (ρ = −0.107, *p* = 3 × 10^−4^) reactions. Mean AE count per patient-year by dose quartile decreased monotonically: Q1 1.59, Q2 1.01, Q3 0.89, Q4 0.65. This dose–AE inverse pattern is most plausibly explained by tolerance development plus survivor selection: patients who tolerated cannabis remained on it and titrated upward, while those experiencing dose-limiting AEs were preferentially down-titrated by the prescribing physician. THC content showed no meaningful correlation with total AE count (ρ = −0.055, *p* = 0.06) or with any individual organ-system AE category. CBD content showed a small positive correlation with total AE count (ρ = +0.137, *p* < 10^−5^), most plausibly reflecting the fact that CBD-rich preparations were preferentially prescribed to patients with greater anxiety, sleep, and fibromyalgia comorbidity, in whom symptom reporting was correspondingly higher. Critically, across all 1205 patient-years, no patient-year contained a moderate or severe (grade ≥ 2) cannabis-related AE, providing the strongest possible reassurance against a dose- or composition-dependent severe-event signal in this cohort. These exposure–AE relationships are reported as descriptive given the observational design and should not be interpreted causally.

### 3.10. Exploratory Ancillary Physiological Indices (Limited Clinical Utility)

BioWell GDV parameters were retained as exploratory ancillary measures with explicitly limited inferential weight (Supplementary Figure S2). The BioWell Stress Activity Coefficient (BWS) and Vitality Energy Potential (BWV) showed non-monotonic trajectories across Years 0–5 that did not parallel the rapid Year-1 PROM improvements. Discrimination for clinically meaningful pain (NRS > 4) was poor: BWS area under the receiver-operating-characteristic curve was 0.49 (95% bootstrap CI 0.46–0.53); BWV (sign-flipped) was 0.55 (95% CI 0.52–0.59)—both at or near chance. The BWS × baseline-anxiety interaction was non-significant (β = −0.13, *p* = 0.22) in cluster-robust OLS, although stratified Pearson correlations (BWS vs. NRS) were modestly stronger in anxiety-negative (r = 0.22) than anxiety-positive (r = 0.12) patients, consistent with our group’s prior ultra-weak photon emission work showing partial specificity for affective–autonomic rather than nociceptive states (Yassin et al. 2025) [14]. These BioWell findings should be regarded as hypothesis-generating only; in agreement with recent independent critiques [16,17], BioWell GDV does not currently meet criteria for a validated pain biomarker.

## 4. Discussion

In this five-year retrospective analysis of prospectively collected outcomes from 241 treatment-refractory CLBP patients, inhaled medical cannabis was associated with large, statistically robust, and clinically meaningful sustained improvements in pain (NRS Δ −5.36), disability (ODI Δ −17.68), pain severity (BPI-S Δ −6.73), and pain interference (BPI-I Δ −3.41). Year-5 retention reached 98.8% for outcome assessment and 92.9% for active cannabis therapy, with only 5/241 patients (2.1%) discontinuing for adverse events or inefficacy. Concomitant medication trajectories showed near-universal displacement of opioids (100% to 4.6%; within-patient ARR 95.4%), NSAIDs (100% to 7.1%), SSRI/SNRIs (80.5% to 5.4%), and gabapentinoids (38.6% to 2.5%); the absolute risk reduction for opioid use vs. the within-patient maximal-conventional-therapy state corresponds to an NNT of 1.05 (we note this is an ARR-derived NNT, not a between-arm randomized contrast). The adverse-event profile was dominated by mild (99.8%) ocular, cognitive, and gastrointestinal events well known to inhaled-cannabis pharmacology.

These findings contribute to the long-term cannabis-pain literature in three ways. First, the cohort’s documented refractoriness—every patient had failed ≥ 1 year of opioids, NSAIDs, and physiotherapy, and the majority had additionally failed antidepressants and anticonvulsants—creates an unusually homogeneous and clinically relevant historical-control comparator. Second, the five-year duration is substantially longer than most observational cannabis cohorts and the great majority of randomized trials, which rarely extend beyond 6–12 months [5,8,9,10,15]. Third, the analytic framework adopted here (MMRM with random slopes, multiple imputation as primary, multivariate Hotelling T^2^, GEE for binary trajectories, Cox time to discontinuation, dose × time interactions) addresses the methodological critiques most frequently leveled at observational pain research.

### 4.1. Comparison with Recent Evidence

Our Year-1 NRS reduction (−3.97 points; from 8.08 to 4.10) is at the upper bound of effects reported in recent randomized trials. The phase-3 VER-01 trial (Karst et al., 2025) demonstrated significant improvements in chronic pain at 12 weeks with full-spectrum THC/CBD extract [10]. The 2021 BMJ rapid-recommendation network meta-analysis (Wang et al.) found small to moderate benefits with high-quality evidence for medical cannabis in chronic pain [5]. The most relevant comparator for the long-term horizon of the present study is the 2025 Lancet Rheumatology systematic review and meta-analysis of long-term effectiveness of non-surgical interventions for chronic low back pain (Jenkins et al., 75 trials, 15,395 participants), which reported that the best-supported non-cannabis interventions—cognitive behavioral therapy, mindfulness, multidisciplinary care, and exercise—produced only small reductions of approximately −7 to −10 points on a 100-point pain or disability scale at long-term follow-up, with moderate-to-low certainty of evidence [11]. The magnitude of Year-1 effects in this refractory cohort exceeds those pooled trial estimates by a factor of roughly five on the equivalent scale, almost certainly reflecting (a) the selection of patients with severe baseline pain (NRS 8.1) who have correspondingly more room to improve, (b) a within-subject design that removes between-patient variance, and (c) the well-documented expectancy effects of an actively chosen and self-titrated therapy. The Year-2-to-Year-5 sustainability of the effect, however, is novel: short-term trials cannot speak to whether early gains are maintained or eroded by tolerance, and the Jenkins meta-analysis found only very limited, very-long-term (≥2 year) evidence for any non-surgical CLBP intervention [11]. The trend-level negative year × dose interaction (β = −0.0044, *p* = 0.074) is consistent with no pharmacological tolerance over the five-year horizon and aligns with the dose-stable, mechanism-based effects observed with other CNS-active analgesics in selected responders. We do not over-interpret a non-significant interaction; the substantively meaningful finding is the absence of a positive interaction (which would have signaled tolerance). We caution that all comparisons against trial estimates above are descriptive across heterogeneous designs and cohorts; the present analysis cannot adjudicate whether the larger effect reflects true differential efficacy, baseline-severity selection, or expectancy bias intrinsic to the open-label observational format.

### 4.2. Concomitant-Medication Sparing as a Clinically Meaningful Endpoint

The opioid-sparing signal in this cohort—a within-patient absolute risk reduction of 95.4% from Year 0 to Year 5, with paired McNemar exact *p* = 1.16 × 10^−69^—is the most clinically actionable finding. We caution that the corresponding ARR-derived NNT of 1.05 is a within-patient estimate referenced to each patient’s own documented maximal-conventional-therapy state and is therefore not equivalent to a between-arm NNT from a randomized comparator trial; the two quantities differ in their reference comparator and should not be directly substituted in cross-study comparisons. Several non-pharmacological factors could nonetheless have contributed to the observed opioid de-escalation, including: (a) the clinical care relationship itself, in which structured annual review may have provided opportunity and motivation for opioid taper independent of any cannabis effect; (b) physician-driven tapering decisions in an open-label single-prescriber context where the prescribing clinician was aware of cannabis initiation and may have actively encouraged opioid reduction; (c) parallel public-health and guideline pressures during 2020–2025 that may have produced secular reductions in long-term opioid use across the Israeli healthcare system; and (d) the inverse possibility—that pain relief from cannabis enabled opioid reduction—which we regard as the most parsimonious explanation but cannot definitively isolate from the alternatives. Nonetheless, the binary fact of stopped opioid prescriptions is mechanically less susceptible to placebo, expectancy, or regression-to-the-mean biases than continuous PROMs, and the magnitude (95% within-patient ARR) substantially exceeds any plausible secular trend. In an era when the opioid epidemic continues to generate substantial morbidity and mortality [2,3], a treatment associated with discontinuation of long-term opioid therapy in 95% of refractory CLBP patients merits serious consideration, even with the inevitable causal-inference caveats of an observational design. The magnitude observed here meaningfully exceeds the typical opioid-cessation rates of 30–60% reported in earlier observational and prospective cannabis cohorts [7,12,13] and the Wang 2021 BMJ rapid-recommendation network meta-analysis of randomized trials [5], which is consistent with our cohort being more uniformly refractory at baseline (every patient with documented opioid failure prior to enrolment) and followed for substantially longer (5 years vs. typically 3–18 months in the comparator literature). The parallel near-elimination of NSAIDs (avoiding cardiovascular and renal toxicity over years of chronic use) and SSRI/SNRIs (avoiding metabolic, sexual, and discontinuation-syndrome harms) extends the public-health relevance.

### 4.3. Telemedicine Attrition and Year-3 Retention Pattern

The non-monotonic retention pattern (100% Y0–Y1 → 96.7% Y2 → 88.8% Y3 → 95.0% Y4 → 98.8% Y5) requires comment because it could superficially be misread as treatment failure or selective drop-out at Year 3. The pattern instead reflects a real-world Israeli health-system phenomenon: between approximately 2022 and early 2024, several telemedicine cannabis-licensure clinics offered convenient phone-based renewals, and a subset of our patients drifted to those clinics during their peak availability while continuing cannabis. After Israeli police action against several of these telemedicine clinics in 2024, those patients returned to in-person follow-up at our center, accounting for the Year-4 and Year-5 retention recovery. None of these patients formally discontinued cannabis; they simply obtained their prescription elsewhere for one or two years. The MMRM framework handles this MAR pattern correctly by borrowing strength from observed Year-2 and Year-4 visits, and the multiple-imputation sensitivity analysis confirmed near-identical effect estimates (FMI < 0.03 for all primary outcomes), making this a methodologically benign artefact.

### 4.4. Exploratory BioWell GDV Findings: Limited Clinical Utility

The BioWell GDV system uses pulsed electric-field-stimulated coronal-discharge imaging at the fingertips to compute composite indices (Activity Coefficient as a stress proxy; Energy Potential as a vitality proxy) [16,17,21,22]. Its physical basis—gas discharge initiated by skin-surface charge accumulation, modulated by electrolyte composition, hydration, and autonomic skin conductance—is uncontroversial in the bioelectrical-impedance and skin-potential literatures. However, the clinical inferential leap from raw GDV imagery to multi-dimensional psychophysiological constructs has been rigorously challenged in recent independent workHowever, the clinical inferential leap from raw GDV imagery to multi-dimensional psychophysiological constructs has been rigorously challenged in recent independent work. Miraglia (2024, Parts I and II) [16,17] documented substantial measurement variability and poor cross-subject reliability of GDV-derived metrics. Our own data are consistent with that critique: BWS and BWV showed non-monotonic year-over-year patterns that did not parallel PROM trajectories, and ROC discrimination of NRS > 4 was at or near chance (BWS AUC 0.49; BWV AUC 0.55). We therefore retain BioWell only as an exploratory ancillary measure, transparently labeled as such, and explicitly disclaim any pain-biomarker interpretation. The modest Pearson r = 0.22 between BWS and NRS in anxiety-negative patients (versus r = 0.12 in anxiety-positive) hints—consistent with our group’s recent ultra-weak photon emission work in cannabis-treated neuropathic pain (Yassin et al. 2025, Bioengineering 12:1359 [14])—that any GDV signal in this domain is probably more closely related to autonomic/affective state than to pain itself, and should be investigated, if at all, in dedicated psychophysiological rather than pain studies.

### 4.5. Strengths

Major strengths of this analysis include: (a) the documented and uniform pre-cannabis multimodal-therapy failure period creating a clinically credible within-subject historical control; (b) a five-year follow-up duration with 98.8% Year-5 retention, exceptional in cannabis observational research; (c) systematic per-organ-system AE recording with severity, causality, and outcome grading across 1205 patient-years; (d) MMRM specification with explicit random-intercept-and-slope structure, REML estimation, convergence verification, and ICC reporting (compliant with FDA/EMA MMRM reporting standards for longitudinal pain trials); (e) multiple imputation (m = 20, Rubin’s rules) as the primary missing-data approach with FMI quantification; (f) multivariate Hotelling T^2^ as a single joint test across the four correlated PROMs, addressing inter-outcome dependence directly; (g) GEE logistic plus exact McNemar test for binary medication trajectories; (h) Kaplan–Meier and Cox time to discontinuation; (i) pre-specified Bonferroni correction at α = 0.0125 for the four co-primary outcomes; and (j) MCID-anchored responder analyses with NNT for direct clinical interpretability.

### 4.6. Limitations

Single-arm, single-center, single-prescriber observational design conducted in one Israeli orthopedic practice: causal inference is constrained, and unmeasured confounding cannot be excluded. The within-subject historical-control design mitigates between-patient confounding but does not address several recognized biases that warrant explicit acknowledgment. (a) Regression to the mean is an inherent threat in any single-arm before-vs-after comparison initiated when symptoms are at their most severe; the cohort entered the study with mean NRS 8.1 (a near-ceiling value), and a portion of the subsequent Year-1 improvement plausibly reflects statistical reversion toward each patient’s long-run mean independent of any pharmacological effect, although the durability of improvement through Years 2–5 argues against regression-to-the-mean as the dominant mechanism. (b) Expectancy and self-selection effects intrinsic to an actively chosen, open-label, self-titrated therapy invite response bias on patient-reported outcomes, particularly NRS, ODI, and BPI subscales. (c) Time-dependent confounding may operate via concurrent changes in physiotherapy use, lifestyle modifications, secular trends in pain reporting, or the supportive clinical relationship itself; while we adjusted for measured baseline characteristics and time-varying dose, unmeasured time-varying confounders remain a possibility. (d) Concomitant-medication discontinuation could theoretically lag rather than parallel clinical improvement, partially confounding interpretation of pain reduction; however, the trajectories of opioid use (steep drop by Year 1) and PROM improvement (largest gains by Year 1) are simultaneous, supporting a treatment-mediated rather than ascertainment-mediated explanation. Binary medication-discontinuation outcomes are mechanically less vulnerable to placebo and expectancy biases than continuous PROMs, but they are not immune to physician-bias effects in an open-label single-prescriber setting. (e) The PROMs are patient-reported and unblinded; an open-label, self-titrated therapy invites response bias. The cohort was treated within the Israeli Ministry of Health medical-cannabis framework, which differs in licensure, formulary, and clinical-care patterns from regulatory environments in the United States, the European Union, the United Kingdom, Canada, and Australia; replication in independent multi-site cohorts in those settings is therefore essential before the present findings should be regarded as broadly generalizable. Approximately 91% of patients used inhaled (smoked) cannabis as the predominant administration route, with vaporization or oral formulations as occasional adjuncts; smoking-dominant findings should not be extrapolated to vaporization-only or oral-formulation protocols, which differ in pharmacokinetics, peak plasma concentration, and respiratory-exposure profile, and which may yield different effect sizes, adverse-event profiles, and retention patterns. The cohort was also racially and ethnically relatively homogeneous, limiting external validity to other populations. Some baseline variables (e.g., precise prior-opioid morphine-milligram-equivalent dose, exact prior gabapentin daily dose) were not available retrospectively. The five-year endpoint, while substantial, cannot speak to lifetime risk of cannabis-use disorder or to safety beyond 5 years. The Year-3 telemedicine attrition, although well explained and methodologically benign, represents a non-random missingness pattern that we addressed via MAR-based multiple imputation; if the true mechanism is partly missing-not-at-random, MI may slightly underestimate uncertainty (the tipping-point pattern-mixture sensitivity analysis indicated that very pessimistic MNAR assumptions did not overturn the primary inference). Finally, our exclusion of patients without prior multimodal-therapy failure means findings should not be extrapolated to opioid-naïve or non-refractory CLBP populations, in whom less aggressive, lower-risk therapies should remain first-line.

### 4.7. Implications and Future Directions

These five-year observational data are consistent with inhaled medical cannabis being a clinically meaningful option in CLBP patients who have failed multimodal conventional therapy, with a benefit–risk profile dominated by large, sustained PROM improvements; near-universal opioid sparing; and predominantly mild, well-characterized adverse events. Confirmation of these associations as causal effects requires randomized comparative trials. Future work should pursue (i) randomized comparative-effectiveness trials of inhaled cannabis versus continued multimodal therapy in refractory CLBP, with longer follow-up than typical short-term cannabis trials; (ii) mechanistic studies linking THC/CBD ratios, dose, and route to pain phenotypes; (iii) implementation studies of opioid-tapering protocols incorporating cannabis as a sparing agent under regulated medical supervision; and (iv) refinement of objective adjunctive measures—if any are clinically informative—for pain-state monitoring, recognizing that BioWell GDV in particular has not in this cohort or in independent studies met validity criteria for that role.

## 5. Conclusions

In a five-year retrospective analysis of 241 treatment-refractory chronic low back pain patients with each patient serving as their own historical control, inhaled medical cannabis was associated with large and durable improvements in pain (NRS Δ −5.36), disability (ODI Δ −17.68), and Brief Pain Inventory severity (Δ −6.73) and interference (Δ −3.41); all four contrasts had |z| ≥ 16.9 and exceeded the Bonferroni-adjusted significance threshold (α = 0.0125) by more than twenty orders of magnitude. The multivariate Hotelling T^2^ joint test across all four correlated PROMs yielded F(4232) = 872.8, *p* < 10^−20^. At Year 5, 89.2% of patients achieved ≥ 30% NRS reduction, 77.2% ≥ 50% reduction, and 93.4% met the NRS minimum clinically important difference. Concomitant opioid use fell from 100% to 4.6% (within-patient ARR 95.4%; McNemar exact *p* = 1.16 × 10^−69^) and NSAID use from 100% to 7.1%, while five-year retention on active cannabis prescription was 92.9% with only 2.1% discontinuing for adverse events or inefficacy. The adverse-event profile was dominated by 99.8% mild ocular, cognitive, and gastrointestinal events; no patient-year contained a moderate or severe (grade ≥ 2) cannabis-related event. Multiple imputation as the primary missing-data approach, MMRM with explicit handling of random-effects singularity, and dose × time analyses confirmed robustness and were consistent with no pharmacological tolerance. Exploratory BioWell GDV indices showed chance-level pain discrimination and are reported only as such, in agreement with recent independent critiques of GDV reliability. These results describe associations rather than causal effects: in the absence of a concurrent randomized control arm, regression-to-the-mean, expectancy and self-selection effects, secular trends, and unmeasured time-dependent confounding cannot be excluded, and the observed improvements likely reflect a combination of pharmacological action and these non-pharmacological factors. Randomized comparative trials of inhaled cannabis versus continued multimodal therapy in refractory CLBP are needed before causal claims can be made. In their absence, and pending such confirmation, these data support consideration of inhaled cannabis as a potentially clinically meaningful, opioid-sparing option for patients who have failed conventional multimodal therapy.

## Figures and Tables

**Table 1:** Baseline characteristics, comorbidities, prior conventional therapy, and pre-cannabis (Year 0) PROMs of the 241-patient refractory CLBP cohort.

Variable	Value (Mean ± SD or n/N (%))	Median (IQR) or Detail
Demographics		
Age (years)	49.3 ± 14.9	50 (40–59)
Female sex	91/241 (37.8%)	
BMI (kg/m^2^)	29.3 ± 6.0	29 (25–33)
Pain duration (years)	15.1 ± 11.2	11 (9–17)
Comorbidity at baseline		
Fibromyalgia	107/241 (44.4%)	
Anxiety	107/241 (44.4%)	
Past psychosis	10/241 (4.1%)	
Sleep disorder	67/241 (27.8%)	
Prior conventional therapy (≥1 y)		
Opioid analgesics	241/241 (100%)	8.6 ± 3.6 y
Gabapentin/pregabalin	93/241 (38.6%)	0.6 ± 1.1 y
SSRI/SNRI	194/241 (80.5%)	1.3 ± 0.8 y
NSAID	241/241 (100%)	7.5 ± 7.6 y
Physiotherapy	241/241 (100%)	7.2 ± 2.9 mo
Baseline (Year 0) PROMs		
NRS pain (0–10)	8.1 ± 1.6	9 (7–9)
ODI (% disability)	55.0 ± 15.9	56 (42–68)
BPI severity (0–10)	7.9 ± 1.7	8 (7–9)
BPI interference (0–10)	5.8 ± 2.0	6 (5–7)
BioWell stress (AC, 0–10)	4.46 ± 1.04	4.50 (3.50–5.40)
BioWell vitality (EP, %)	50.3 ± 7.8	50 (45–55)

**Table 2:** Primary MMRM results: Year-5 minus Year-0 changes in the four pre-specified primary PROMs (categorical time, random intercept + slope, REML), with multiple-imputation (m = 20, Rubin’s rules) pooled estimates and Bonferroni-adjusted significance threshold α = 0.0125.

PROM	Y0 Mean (95% CI)	Y5 Mean (95% CI)	Δ Y5–Y0 (95% CI)	MI-Pooled Δ (95% CI)	*p*-Value
NRS	8.08 (7.78, 8.38)	2.71 (2.48, 2.95)	−5.36 (−5.65, −5.07)	−5.37 (−5.66, −5.07)	<10^−20^
ODI	55.05 (52.63, 57.46)	37.36 (35.90, 38.81)	−17.68 (−19.73, −15.63)	−17.63 (−19.66, −15.60)	<10^−20^
BPI severity	7.94 (7.69, 8.19)	1.20 (1.03, 1.37)	−6.73 (−6.99, −6.47)	−6.73 (−6.99, −6.47)	<10^−20^
BPI interference	5.84 (5.62, 6.07)	2.44 (2.20, 2.68)	−3.41 (−3.65, −3.16)	−3.41 (−3.66, −3.16)	<10^−20^

**Table 3:** Year-5 responder rates and number needed to treat (NNT) for medication discontinuation.

Endpoint	n/N	Year-5 Rate	NNT
NRS ≥ 30% reduction	212/238	89.2%	—
NRS ≥ 50% reduction	184/238	77.2%	—
NRS MCID (≥2 pts or ≥30%)	222/238	93.4%	—
ODI MCID (≥10 pts or ≥30%)	156/238	65.6%	—
BPI-S MCID (≥1 pt)	233/237	98.3%	—
BPI-I MCID (≥1 pt)	216/237	91.3%	—
Opioid discontinuation Y5	230/241	95.4% ARR	1.0
NSAID discontinuation Y5	224/241	92.9% ARR	1.1

NNT, number needed to treat; ARR, absolute risk reduction; MCID, minimum clinically important difference. “—” denotes not applicable: NNT is reported only for binary within-patient medication-discontinuation outcomes referenced to each patient’s documented pre-cannabis maximal-conventional-therapy state, and is not defined for continuous PROM responder-rate endpoints. The within-patient ARR-derived NNT shown for opioid and NSAID discontinuation is not equivalent to a between-arm randomized-trial NNT.

**Table 4:** GEE-modeled marginal probabilities (with 95% CIs) of active concomitant therapy at each follow-up year, with exact McNemar tests for opioid and NSAID transitions.

Therapy	Y0	Y1	Y2	Y3	Y4	Y5
Opioid	100%	22.8%	22.8%	19.5%	1.7%	4.6%
Gabapentinoid	38.6%	45.2%	1.7%	1.7%	2.1%	2.5%
SSRI/SNRI	80.5%	4.6%	4.6%	6.6%	4.6%	5.4%
NSAID	100%	11.6%	3.3%	4.6%	2.9%	7.1%
Physiotherapy	4.1%	12.0%	13.3%	14.1%	13.3%	17.0%
Other CAM	42.3%	47.7%	34.4%	34.4%	17.4%	24.1%

## Data Availability

De-identified data and analysis code are available from the corresponding author on reasonable request and subject to data-sharing agreements consistent with the IRB protocols and Israeli Ministry of Health regulations.

## Supplementary Materials

The following supporting information can be downloaded at: https://www.mdpi.com/article/10.3390/biomedicines14061255/s1, Table S1: Random-effects diagnostics, Table S2: Descriptive statistics by year, Table S3: Adverse-event rates per organ system, Table S4: McNemar exact tests for medication discontinuation, Figure S1: Kaplan–Meier retention curves, Figure S2: BioWell GDV ROC analysis. The complete reproducible analysis pipeline is supplied as Supplementary Addendum 1 (analysis_v6.py).
