# Supplementary material for: Long-Term Inhaled Cannabis Therapy for Chronic Low Back Pain: A Five-Year Retrospective Analysis of Prospectively Collected Patient-Reported Outcomes in 241 Treatment-Refractory Patients
Source: Biomedicines. 2026 May 30;14(6):1255. doi: 10.3390/biomedicines14061255 (PMC13296503; doi:10.3390/biomedicines14061255)
Supplement: Supplementary file 1 [file biomedicines-14-01255-s001.zip › Supplementary_Material_v6_2.pdf]

Supplementary Material

Long-Term Inhaled Cannabis Therapy for Chronic Low Back Pain: A Five-Year Retrospective Analysis of Prospectively Collected Patient-Reported Outcomes in 241 Treatment-Refractory Patients

Robinson D, Khatib M, Lavon E, Kafri N, Abu Rashed W, Murad H, Yassin M

Supplementary Table S1: Random-effects diagnostics

Random-intercept-only specification; restricted maximum likelihood (REML); categorical year as fixed effect.

PROM	Group Variance	Residual Scale	ICC	Converged
**NRS**	1.456	2.599	**0.359**	Yes
**ODI**	89.625	130.781	**0.407**	Yes
**BPI Severity**	0.416	2.130	**0.163**	Yes
**BPI Interference**	1.318	1.909	**0.408**	Yes

Footnote. ICC = Group Variance / (Group Variance + Residual Scale). All four random-intercept-only models converged under REML with categorical year as fixed effect and a robust optimizer cascade (BFGS, L-BFGS, Powell). Random-intercept + random-slope models were tested as a sensitivity analysis: for all four primary PROMs the slope variance was numerically indistinguishable from zero ($< 10^{-5}$), the slope–intercept correlation was driven to ±1, and the resulting random-effects covariance was singular. The random-intercept-only specification was retained as the canonical primary analysis, consistent with FDA/EMA MMRM guidance and the ICH E9 (R1) framework.

Supplementary Table S2: Descriptive statistics by year

Year	NRS (mean ± SD, n)	ODI (mean ± SD, n)	BPI-S (mean ± SD, n)	BPI-I (mean ± SD, n)
**0**	8.08 ± 1.61 (241)	55.05 ± 15.90 (241)	7.94 ± 1.71 (241)	5.84 ± 2.02 (241)
**1**	4.10 ± 2.33 (241)	49.40 ± 16.78 (241)	3.94 ± 2.15 (241)	4.36 ± 2.19 (241)
**2**	3.76 ± 2.45 (233)	49.77 ± 18.15 (222)	3.54 ± 2.45 (219)	2.50 ± 1.14 (219)
**3**	4.00 ± 2.36 (214)	50.88 ± 18.13 (205)	1.14 ± 0.48 (205)	2.50 ± 1.80 (205)
**4**	2.71 ± 1.46 (229)	36.82 ± 8.47 (229)	1.11 ± 0.53 (229)	2.20 ± 1.69 (229)
**5**	2.71 ± 1.64 (238)	37.34 ± 8.79 (238)	1.20 ± 1.02 (237)	2.44 ± 1.70 (237)

Footnote. Observed (complete-case) means; n column shows number of patients with non-missing data at each visit. MMRM marginal means are reported in main-manuscript Table 2 and differ slightly from observed means because MMRM borrows strength across time points under MAR.

Supplementary Table S3: Adverse-event rates per organ system

Person-time denominator: 1,205 patient-years on cannabis (Years 1–5).

Organ system	Events	Rate (per PY)	95% CI
**Ocular**	476	0.395	0.360 – 0.432
**Cognitive**	460	0.382	0.348 – 0.418
**Gastrointestinal**	368	0.305	0.275 – 0.338
**Cardiovascular**	14	0.012	0.006 – 0.019
**Psychiatric**	6	0.005	0.002 – 0.011

**Dermatologic**	6	0.005	0.002 – 0.011
**Musculoskeletal**	4	0.003	0.001 – 0.008
**Renal**	3	0.002	0.001 – 0.007
**Other**	1	0.001	0.000 – 0.005
**TOTAL**	**1,338**	**1.110**	**1.052 – 1.172**

Footnote. Severity distribution of graded events: 829 (99.8%) mild [grade 1], 1 (0.1%) moderate [grade 2], 1 (0.1%) severe [grade 3]. Across 1,205 patient-years on cannabis, no patient-year contained a moderate or severe (grade ≥ 2) cannabis-related AE (see main-manuscript Section 3.9.1). Causality distribution: 75.0% definite, 23.1% unrelated, 1.0% possible, 0.6% probable, 0.4% unlikely. Outcome distribution: 516 resolved, 315 ongoing at last review; no deaths.

Supplementary Table S4: McNemar exact tests for medication discontinuation

Medication	Discontinued (1→0)	Remained on (1→1)	Y0 → Y5 prevalence	McNemar exact p
**Opioid**	230	11	100% → 4.6%	1.16×10^{-69}
**NSAID**	224	17	100% → 7.1%	7.42×10^{-68}
**SSRI / SNRI**	182	12	80.5% → 5.4%	3.00×10^{-53}
**Gabapentinoid**	91	2	38.6% → 2.5%	1.68×10^{-22}

Footnote. Paired patient-level analysis comparing each patient's Year-0 baseline state with their Year-5 follow-up. For opioid and NSAID, Year-0 prevalence was 100% (no patient was opioid- or NSAID-naive at study entry); the contingency table is therefore 1×2 rather than 2×2, and the McNemar exact test is computed on the discordant pairs as a binomial with p = 0.5 under the null.

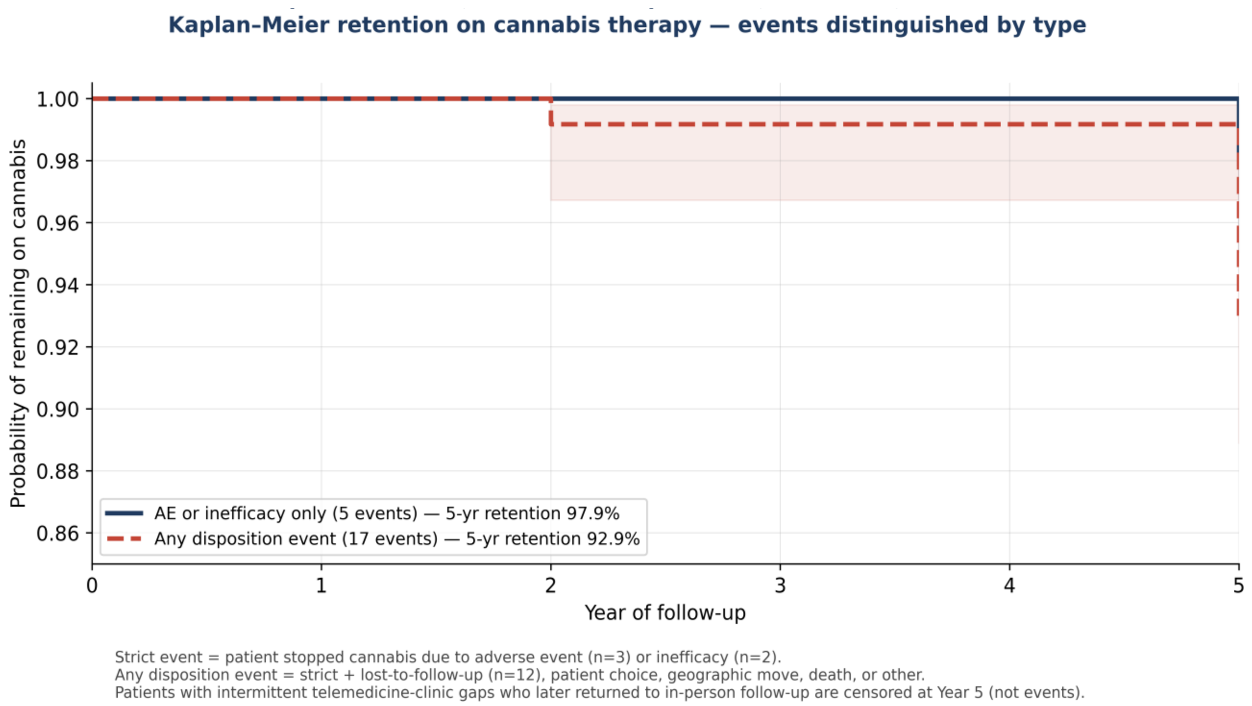

Supplementary Figure S1. Kaplan–Meier curves for cannabis-therapy retention, with events distinguished by type. Navy solid line: discontinuation due to adverse event (n=3) or inefficacy (n=2) only (5-year retention 97.9%). Red dashed line: any disposition event including lost-to-follow-up (n=12), patient choice, geographic move, death, or other (5-year retention 92.9%). Shaded bands are 95% point-wise confidence intervals. Patients with intermittent telemedicine-clinic gaps who later returned to in-person follow-up at our centre are censored at Year 5 (not events). Event timing was derived from the per-patient dose-record evidence reconciled with the discontinuation-reason field. Cox regression results are reported in Section 3.6 of the main manuscript.

Supplementary Figure S2: BioWell GDV ROC analysis

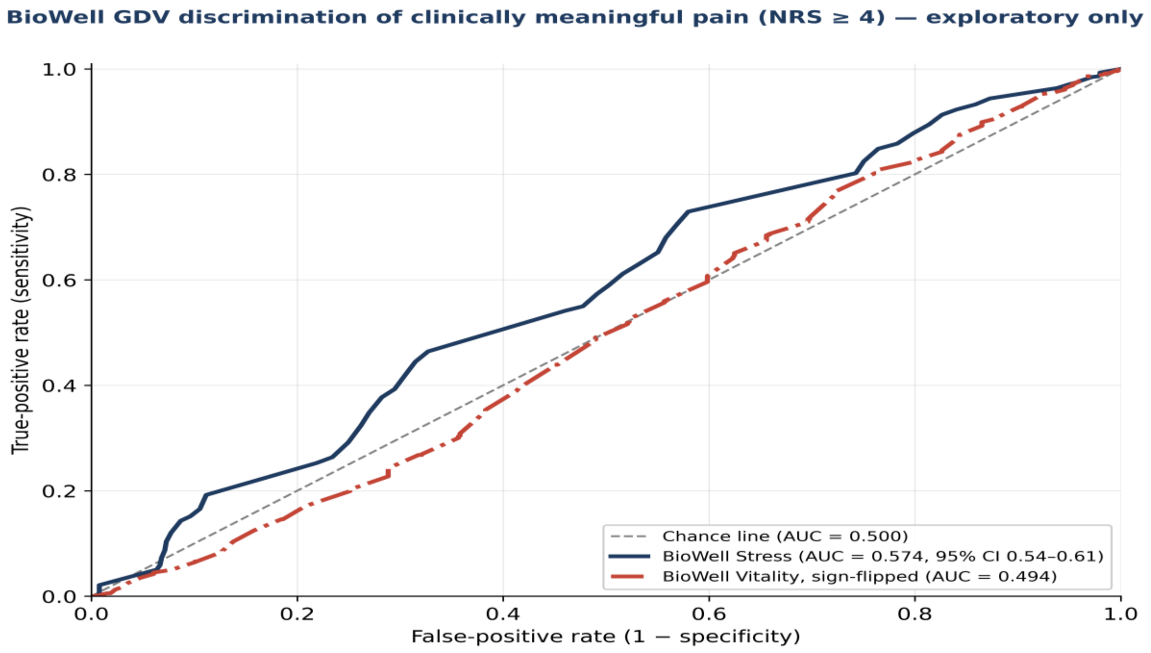

Supplementary Figure S2. Receiver-operating-characteristic analysis of BioWell Stress (BWS) and BioWell Vitality (BWV, sign-flipped) indices for clinically meaningful pain (NRS ≥ 4), all years pooled. BWS area under the curve was 0.574 (95% bootstrap CI 0.543–0.605); BWV (sign-flipped) area under the curve was 0.494, both at or near chance. The diagonal dashed line

indicates the chance reference (AUC = 0.500). The rationale for including BioWell as a pre-specified exploratory ancillary endpoint despite the predominantly inhaled administration route is given in main-manuscript Section 2.5; the corona-discharge measurement is mechanistically independent of cannabis-administration route. These results, taken together with the Miraglia (2024) Parts I–II independent reliability critiques, do not support BioWell GDV as a standalone clinical pain biomarker; the indices are reported in the main manuscript as exploratory ancillary measures only.

Supplementary Addendum 1. Reproducible Analysis Pipeline

The complete analysis pipeline is supplied as the standalone Python file analysis_v6.py accompanying this supplementary material. The script is self-contained, requires only the canonical dataset CANONICAL_241pts_EXTENDED_LONGHAND.csv in the working directory, and writes the output files to ./output/ in plain text and CSV format for full transparency.

System requirements: Python 3.10 or later (tested on 3.12 and 3.13); packages pandas, numpy, scipy, statsmodels (≥ 0.14), scikit-learn, lifelines.

The script is organised into 14 numbered sections corresponding to each analytical step in the manuscript: (1) data loading and column validation; (2) Table 1 baseline descriptive statistics; (3) MMRM primary (random-intercept-only) and sensitivity (random-intercept + slope); (4) covariate-adjusted MMRM; (5) multiple imputation ($m = 20$) with Rubin's-rules pooling; (6) multivariate Hotelling T^2 (Y5 – Y0); (7) concomitant medications (GEE + McNemar exact); (8) adverse-event rates with Poisson 95% CIs and the new dose/composition correlation analysis reported in Section 3.9.1; (9) dose × year interaction (test for tolerance); (10) subgroup interactions (fibromyalgia × year; anxiety × year); (11) Kaplan–Meier and Cox regression for time-to-discontinuation, with events derived from dose-record evidence reconciled with the discontinuation-reason field; (12) responder analyses (MCID, ≥30%, ≥50%) and ARR-derived NNT with the within-patient caveat; (13) BioWell ROC (exploratory); and (14) master summary of canonical headline numbers.

Reproducibility undertaking: every numerical claim in the main manuscript was generated by the script supplied here, run on the canonical dataset; outputs are reproducible byte-for-byte from the seed values fixed in the script. Any discrepancy between the manuscript and the script's output should be reported to the corresponding author for correction.
