# Supplementary figures and images for: Long-Term Inhaled Cannabis Therapy for Chronic Low Back Pain: A Five-Year Retrospective Analysis of Prospectively Collected Patient-Reported Outcomes in 241 Treatment-Refractory Patients
Source: Biomedicines. 2026 May 30;14(6):1255. doi: 10.3390/biomedicines14061255 (PMC13296503; doi:10.3390/biomedicines14061255)

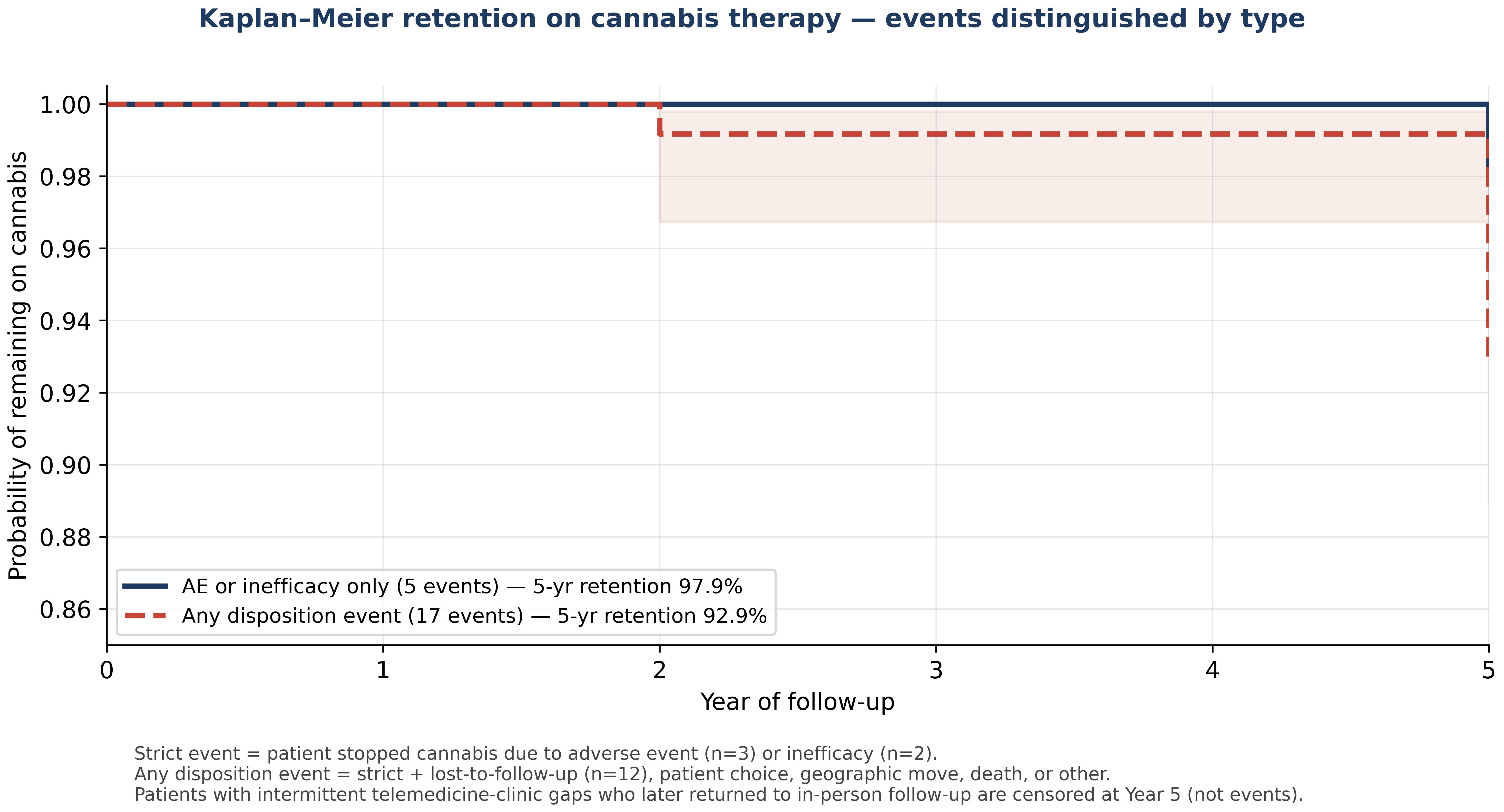

Supplement: Supplementary file 1 [file biomedicines-14-01255-s001.zip › SuppFigS1_KM.png]

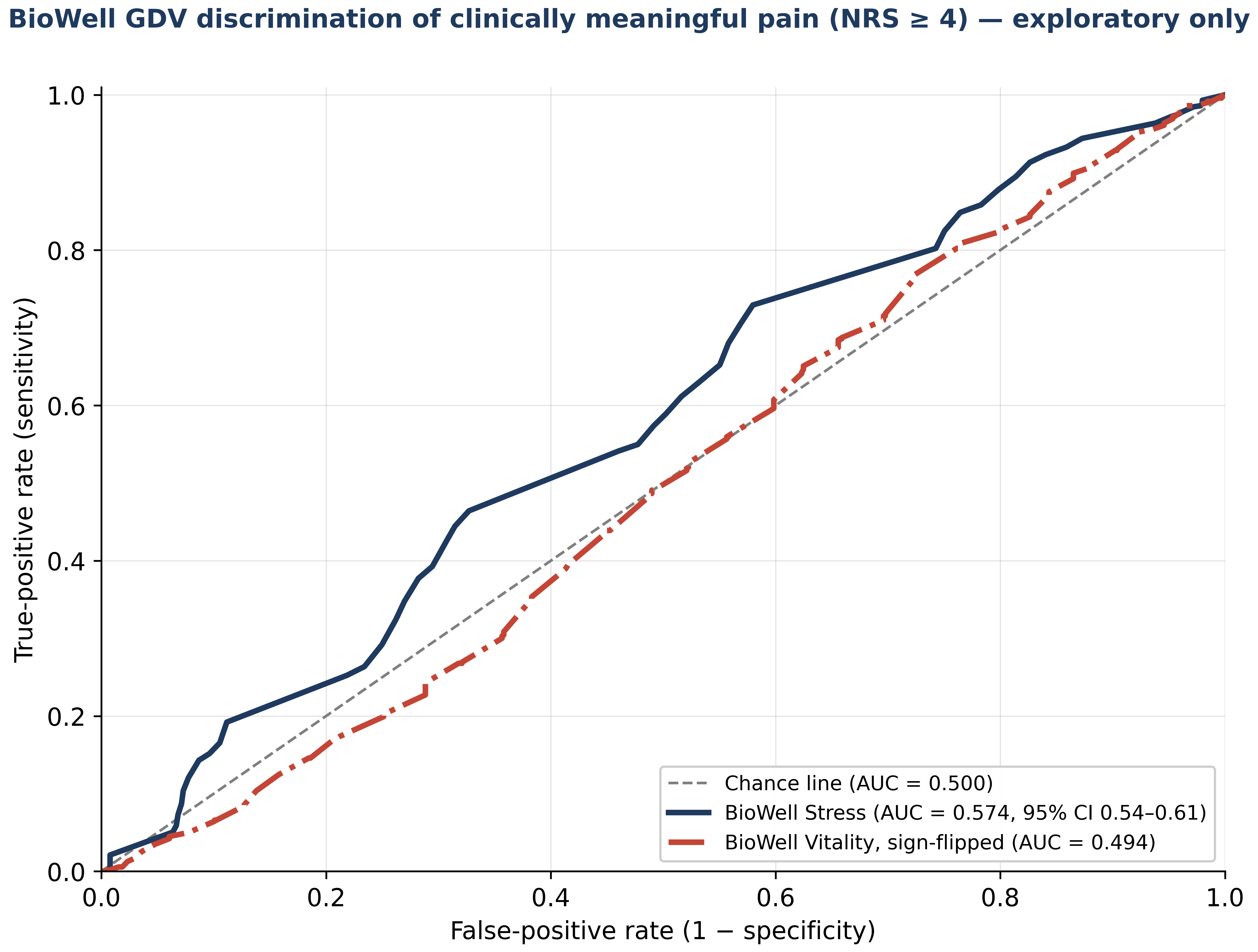

Supplement: Supplementary file 1 [file biomedicines-14-01255-s001.zip › SuppFigS2_BioWell.png]
